# Real-world epidemiology and renal outcomes of ADPKD in Türkiye: the PKD-TURK cohort

**DOI:** 10.1186/s12882-026-05217-0

**Published:** 2026-07-24

**Authors:** Serpil Muge Deger, Necmi Eren, Sibel Yucel Kocak, Gulay Ulusal Okyay, Beyhan Güvercin, Abdulmecit Yildiz, Halime Soyak Kabaca, Sabahat Alisir Ecder, Tahsin Karaaslan, Ayca Inci, Mehmet Asi Oktan, Mine Sebnem Karakan, Asil Demirezen, Ulver Boztepe Derici, Bartu Ediz, Ilhan Kurultak, Bulent Huddam, Ozlem Usalan, Ilker Atay, Aysun Toraman, Haci Hasan Yeter, Kursad Onec, Alper Azak, Kenan Turgutalp, Belda Dursun, Onur Tunca, Sinan Kazan, Tulin Akagun, Saliha Yıldırım, Emre Yasar, Hulya Colak, Hayri Ustun Arda, Selma Alagoz, Hamad Dheir, Musa Pinar, Ramazan Danis, Eren Eynel, Berna Yelken, Gursel Yildiz, Funda Sari, Serkan Bakirdogen, Bulent Kaya, Ozgur Can, Melike Betul Ogutmen, Metin Ergul, Sibel Gokcay Bek, Tevfik Ecder

**Affiliations:** 1 Division of Nephrology, Department of Internal Medicine, Faculty of Medicine, Dokuz Eylul University, İzmir, Türkiye; 2 Division of Nephrology, Department of Internal Medicine, Kocaeli University Faculty of Medicine, Kocaeli, Türkiye; 3 Division of Nephrology, Department of Internal Medicine, Faculty of Medicine, Bakirkoy Dr. Sadi Konuk Training and Research Hospital, University of Health Sciences, Istanbul, Türkiye; 4 Division of Nephrology, Department of Internal Medicine, Faculty of Medicine, Etlik City Hospital, Health Sciences University, Ankara, Türkiye; 5 Division of Nephrology, Department of Internal Medicine, Faculty of Medicine, Karadeniz Technical University, Trabzon, Türkiye; 6 Division of Nephrology, Department of Internal Medicine, Faculty of Medicine, Bursa Uludağ University, Bursa, Türkiye; 7 Division of Nephrology, Department of Internal Medicine, İstanbul Medeniyet University Faculty of Medicine, İstanbul, Türkiye; 8 Division of Nephrology, Department of Internal Medicine, Göztepe Prof. Dr. Süleyman Yalçın City Hospital, Istanbul, Türkiye; 9 Division of Nephrology, Department of Internal Medicine, Akdeniz University Faculty of Medicine, Antalya, Türkiye; 10 Division of Nephrology, Department of Internal Medicine,Faculty of Medicine, Ankara Yıldırım Beyazıt University, Ankara, Türkiye; 11 Division of Nephrology, Department of Internal Medicine, Gazi University Faculty of Medicine, Ankara, Turkey; 12 Division of Nephrology, Department of Internal Medicine, Kartal training hospital, Istanbul, Türkiye; 13 Division of Nephrology, Department of Internal Medicine, Faculty of Medicine, Trakya University, Tekirdag, Türkiye; 14 Division of Nephrology, Department of Internal Medicine, Faculty of Medicine, Mugla Sıtkı Kocman University, Mugla, Türkiye; 15 Division of Nephrology, Department of Internal Medicine, Gaziantep University Faculty of Medicine, Gaziantep, Türkiye; 16 Division of Nephrology, Department of Internal Medicine, Celal Bayar University Faculty of Medicine, Manisa, Türkiye; 17 Division of Nephrology, Department of Internal Medicine, Faculty of Medicine, Hacettepe University, Ankara, Türkiye; 18 Division of Nephrology, Department of Internal Medicine, Faculty of Medicine, Duzce University, Duzce, Türkiye; 19 Division of Nephrology, Department of Internal Medicine, Faculty of Medicine, University of Health Sciences, Balikesir Ataturk Education and Research Hospital, Balıkesir, Türkiye; 20 Division of Nephrology, Department of Internal Medicine, Faculty of Medicine, Mersin University, Mersin, Türkiye; 21 Division of Nephrology, Department of Internal Medicine,Faculty of Medicine, Pamukkale University, Denizli, Türkiye; 22 Division of Nephrology, Department of Internal Medicine, Faculty of Medicine, University of Health Sciences, Afyonkarahisar Education and Research Hospital, Afyonkarahisar, Türkiye; 23 Division of Nephrology, Department of Internal Medicine, Giresun University Faculty of Medicine, Giresun, Türkiye; 24 Division of Nephrology, Department of Internal Medicine, Ankara University Faculty of Medicine, Ankara, Turkey; 25 Division of Nephrology, Department of Internal Medicine, Faculty of Medicine, İzmir City Hospital, Health Sciences University, İzmir, Türkiye; 26 Division of Nephrology, Department of Internal Medicine, Faculty of Medicine, Bagcilar Training and Research Hospital, University of Health Sciences, Istanbul, Türkiye; 27 Division of Nephrology, Department of Internal Medicine, Sakarya University Faculty of Medicine, Sakarya, Türkiye; 28 Nephrology Clinic, Gazi Yaşargil Educational Research Hospital, Diyarbakır, Türkiye; 29 Division of Nephrology, Department of Internal Medicine, Basaksehir Çam ve Sakura City Hospital, Healty Sciences University, İstanbul, Türkiye; 30 Division of Nephrology, Department of Internal Medicine, Canakkale Onsekiz Mart University Faculty of Medicine, Çanakkale, Türkiye; 31 Division of Nephrology, Department of Internal Medicine, Faculty of Medicine, Cukurova University, Adana, Türkiye; 32 Division of Nephrology, Department of Internal Medicine, Haydarpaşa Numune Training and Research Hospital, Healty Sciences University, İstanbul, Türkiye; 33 Department of Nephrology, Liv Hospital Vadi Istanbul, İstanbul, Türkiye

**Keywords:** Autosomal dominant polycystic kidney disease, Epidemiology, Chronic kidney disease, Renal progression, Türkiye

## Abstract

**Introduction:**

Real-world epidemiological and longitudinal outcome data on autosomal dominant polycystic kidney disease (ADPKD) remain limited in underrepresented populations, including Türkiye. We aimed to characterize the epidemiology, regional distribution, and renal outcomes of ADPKD in a large multicenter national cohort.

**Methods:**

In this retrospective multicenter study, adult patients with ADPKD were identified across tertiary nephrology centers in six regions of Türkiye (2010–2025). Study-based prevalence was calculated using Turkish Statistical Institute population data. Longitudinal renal outcomes were evaluated using serial eGFR measurements and Cox regression models identified predictors of ≥ 40% eGFR decline.

**Results:**

A total of 2361 patients were included (mean age 38.6 ± 14 years; 51.8% female). Median province-level study-based prevalence was 3.86 per 100,000 population (IQR 2.92–5.49), with substantial regional variation, highest in the Black Sea region (13.46 per 100,000). Study-based prevalence increased significantly over time (annual percent change 14.4%; 95% CI 13.1–15.6; *p* < 0.001). During a median follow-up of 6 years, 23.9% experienced a ≥ 40% eGFR decline and 10.5% progressed to kidney failure (eGFR < 15 ml/min/1.73m2). Median annual eGFR decline was − 3.33 mL/min/1.73 m²/year. In multivariable analysis, age (adjusted HR 1.02; 95% CI 1.00–1.03; p = 0.010) and hypertension (adjusted HR 2.33; 95% CI 1.25–4.32; p = 0.007) were independently associated with renal progression, whereas tolvaptan use was associated with a lower risk of progression (adjusted HR 0.56; 95% CI 0.37–0.85; p = 0.004). The Mayo Imaging Classification remained independently predictive of renal outcomes (p = 0.027).

**Conclusions:**

This large real-world multicenter cohort highlights the geographically heterogeneous study-based burden of ADPKD across participating regions of Türkiye. Increasing prevalence over time likely reflects improved disease recognition, whereas the relatively low study-based prevalence suggests persistent under-recognition of ADPKD. These findings provide important longitudinal outcome data from an underrepresented population and support the need for earlier detection and improved risk stratification in ADPKD.

**Supplementary Information:**

The online version contains supplementary material available at https://doi.org/10.1186/s12882-026-05217-0.

## Key learning points

What was known

• Epidemiological data on autosomal dominant polycystic kidney disease (ADPKD) are limited in many regions, including Türkiye, with most estimates derived from registry-based or single-center studies.

• While disease progression is well characterized in clinical trials, real-world data integrating epidemiology and longitudinal renal outcomes remain scarce.

This study adds

• This is the first comprehensive, large-scale, real-world assessment of ADPKD epidemiology and clinical outcomes across Türkiye.

• The observed prevalence was substantially lower than expected, suggesting significant underdiagnosis, and a rising temporal trend.

• Tolvaptan use and Mayo Imaging Classification were independently associated with renal outcomes, supporting their prognostic and therapeutic relevance in routine clinical practice.

Potential impact

• These findings highlight the need for improved early detection strategies and standardized diagnostic approaches for ADPKD in routine clinical practice.

• The marked regional variability underscores disparities in healthcare access and disease recognition, informing public health planning and resource allocation.

• Integration of risk stratification tools and disease-modifying therapies may improve long-term renal outcomes in patients with ADPKD.

**Supplementary Information:**

The online version contains supplementary material available at https://doi.org/10.1186/s12882-026-05217-0.

## Introduction

Chronic kidney disease (CKD) remains a major global health challenge, with a substantial and increasing burden worldwide [1]. Türkiye is among the countries with a high CKD burden, with an estimated median prevalence of 11.32% (IQR 10.56–12.07) according to the International Society of Nephrology Global Kidney Health Atlas [2]. Autosomal dominant polycystic kidney disease (ADPKD), the most common hereditary kidney disease in adults, is characterized by progressive cyst development, bilateral kidney enlargement, and gradual decline in kidney function [3, 4]. Although ADPKD typically manifests during the third to fourth decades of life, disease progression is highly heterogeneous and frequently culminates in kidney failure [4].

Globally, ADPKD affects approximately 13 million individuals and represents a major cause of hereditary kidney failure [3]. Reliable epidemiological data are essential for improving disease recognition, healthcare planning, and risk stratification. However, substantial discrepancies persist between genetically predicted prevalence and clinically recognized disease burden. While population-based genetic studies estimate a prevalence ranging from 1 in 500 to 1 in 2,500 individuals [5], clinically ascertained prevalence estimates are considerably lower, generally ranging from 25 to 68 per 100,000 population [3, 4]. This discrepancy underscores the substantial burden of underdiagnosis, with many affected individuals remaining unrecognized until later stages of the disease.

Despite increasing global interest in ADPKD, epidemiological data from several regions, including Türkiye, remain limited. The CREDIT study is the only nationwide study evaluating CKD burden in Türkiye [6], did not provide disease-specific estimates for ADPKD. Existing evidence is largely restricted to single-center experiences or retrospective cohorts, limiting the ability to assess nationwide disease burden, regional variability, and longitudinal clinical outcomes. Registry-based reports indicate that ADPKD accounts for approximately 3% of end-stage kidney disease (ESKD) cases in Türkiye [7], comparable to rates reported in European cohorts [8]. However, these data predominantly reflect advanced disease stages and provide limited insight into earlier-stage disease, regional epidemiological patterns, and long-term renal outcomes in the Turkish population.

Therefore, we aimed to characterize the real-world epidemiology, regional distribution, and renal outcomes of ADPKD in a large multicenter cohort across Türkiye. In addition, we sought to evaluate longitudinal kidney disease progression and identify factors associated with adverse renal outcomes to provide clinically relevant insights into disease burden and risk stratification in an underrepresented population.

## Methods

### Study design and population

This retrospective multicenter cohort study included adult patients with ADPKD followed at tertiary nephrology centers across six geographical regions of Türkiye between January 2010 and June 2025. Participating centers were selected to provide broad national geographic representation.

The study was approved by the Dokuz Eylül University Non-Interventional Clinical Research Ethics Committee (Approval No. 2025/03–22) and conducted in accordance with the Declaration of Helsinki. Owing to the retrospective design, the requirement for informed consent was waived by the Dokuz Eylul University Non-Interventional Clinical Research Ethics Committee.

### Case definition and data collection

Adult patients (≥ 18 years) with ADPKD were identified using the International Classification of Diseases, 10th Revision (ICD-10) code Q61.2 retrieved from institutional electronic health records [9]. Whenever available, clinical and imaging data were reviewed to confirm the diagnosis and exclude alternative cystic kidney diseases. Genetic testing results (*PKD1*/*PKD2*) and additional imaging findings were incorporated when accessible.

Demographic characteristics, comorbidities, medication use, and laboratory data were extracted from electronic medical records. Comorbid conditions included diabetes mellitus, hypertension, and cardiovascular disease. Medication exposure included renin–angiotensin system (RAS) blockers and tolvaptan.

Laboratory data were collected annually starting from the time of first visit. Renal function was assessed using estimated glomerular filtration rate (eGFR) calculated according to the Chronic Kidney Disease Epidemiology Collaboration (CKD-EPI) equation, including updated race-free equations where applicable [10, 11]. Proteinuria was defined as a urine protein-to-creatinine ratio > 0.3. Chronic kidney disease stages were classified according to Kidney Disease: Improving Global Outcomes (KDIGO) guidelines as follows: G1 (≥ 90), G2 (60–89), G3a (45–59), G3b (30–44), G4 (15–29), and G5 (< 15 mL/min/1.73 m²) [12].

### Disease severity assessment

Disease severity was assessed using the Mayo Imaging Classification based on age and height-adjusted total kidney volume (htTKV), categorizing patients into classes 1 A–1E [13]. Higher Mayo classes indicate an increased risk of rapid kidney disease progression and have been validated as predictors of renal outcomes in ADPKD [13, 14].

### Epidemiological analyses

Study-based prevalence estimates were calculated using annual population data obtained from the Turkish Statistical Institute (TÜİK) [15]. Because participating centers did not capture all patients within each geographical region, prevalence analyses were performed using a study-based approach rather than a true population-based design.

Province-level prevalence was calculated by dividing the number of identified ADPKD cases by the population of the corresponding provinces represented in the cohort and expressed per 100,000 population. Provinces without identified cases were assigned a prevalence value of zero.

Regional prevalence analyses were similarly conducted using only provinces represented by participating centers. Provinces were grouped according to six predefined geographical regions of Türkiye, and prevalence estimates were calculated using the aggregate population of included provinces within each region. This approach was adopted to avoid overestimation or underestimation associated with extrapolation to non-represented populations.

Geographic visualization was performed using a choropleth map based on province-level administrative boundaries, with color intensity reflecting the calculated prevalence.

Annual crude prevalence and incidence rates were calculated using diagnosis-year data. Incidence was defined as newly identified ADPKD cases per calendar year, whereas prevalence included cumulative identified cases from the year of diagnosis onward. Temporal trends were assessed using log-linear regression analyses, and annual percent change (APC) values with 95% confidence intervals (CIs) were estimated.

Age- and sex-specific prevalence rates were calculated using TÜİK population distributions for the included provinces. Age groups were allocated in 15-year increments.

### Renal outcomes

Longitudinal renal outcomes were evaluated using serial eGFR measurements obtained between 2010 and 2025 or until the last available follow-up for censored individuals. The first available eGFR measurement was defined as baseline kidney function.

Renal progression endpoints included ≥ 30%, ≥ 40%, and ≥ 50% decline in eGFR, as well as progression to kidney failure defined as eGFR < 15 mL/min/1.73 m². Annual eGFR decline rates were calculated from baseline to the last available follow-up measurement. Time-to-event analyses were performed from baseline to the first occurrence of each renal endpoint.

### Statistical analyses

Statistical analyses were performed using IBM SPSS Statistics version 24 (IBM Corp., Armonk, NY, USA). Continuous variables are presented as mean ± standard deviation or median (interquartile range [IQR]) according to distribution, whereas categorical variables are presented as frequencies and percentages.

Group comparisons were performed using Student’s t-test or Mann–Whitney U test for continuous variables and chi-square or Fisher’s exact tests for categorical variables, as appropriate. A two-sided p value < 0.05 was considered statistically significant.

Predictors of renal progression were evaluated using Cox proportional hazards regression analyses with ≥ 40% decline in eGFR as the primary renal endpoint, a validated surrogate outcome for clinically meaningful kidney disease progression in CKD and ADPKD [13, 16]. Although alternative thresholds such as 30% or 50% decline may also be used, the 40% threshold has been shown to provide a robust and clinically meaningful measure of disease progression while minimizing misclassification of short-term variability. The following variables were analyzed in univariate models: age, sex, body mass index (BMI), genotype, diabetes mellitus, hypertension, cardiovascular disease, use of RAS blockers, tolvaptan use, and Mayo Imaging Classification. Categorical variables were entered using indicator (dummy) coding, with predefined reference categories (female sex, *PKD1* genotype, absence of comorbidities, no treatment exposure, and Mayo class 1 A).

Variables with clinical relevance or *p* < 0.05 in univariate analysis were included in multivariable models using the enter method. The results are reported as hazard ratios (HRs) with 95% confidence intervals (CIs). Multicollinearity was assessed using variance inflation factors (VIFs). Missing data were handled using a complete-case analysis approach.

## Results

### Study population and epidemiological characteristics

The PKD-TURK cohort included 2361 adult patients with ADPKD identified between January 2010 and June 2025 across 32 tertiary nephrology centers located in 21 provinces representing six geographical regions of Türkiye. Patient distribution broadly paralleled regional population density, with the largest proportion of patients originating from the Marmara (51%), Aegean (15%), and Central Anatolia (13%) regions (Table 1).

**Fig. 1:**
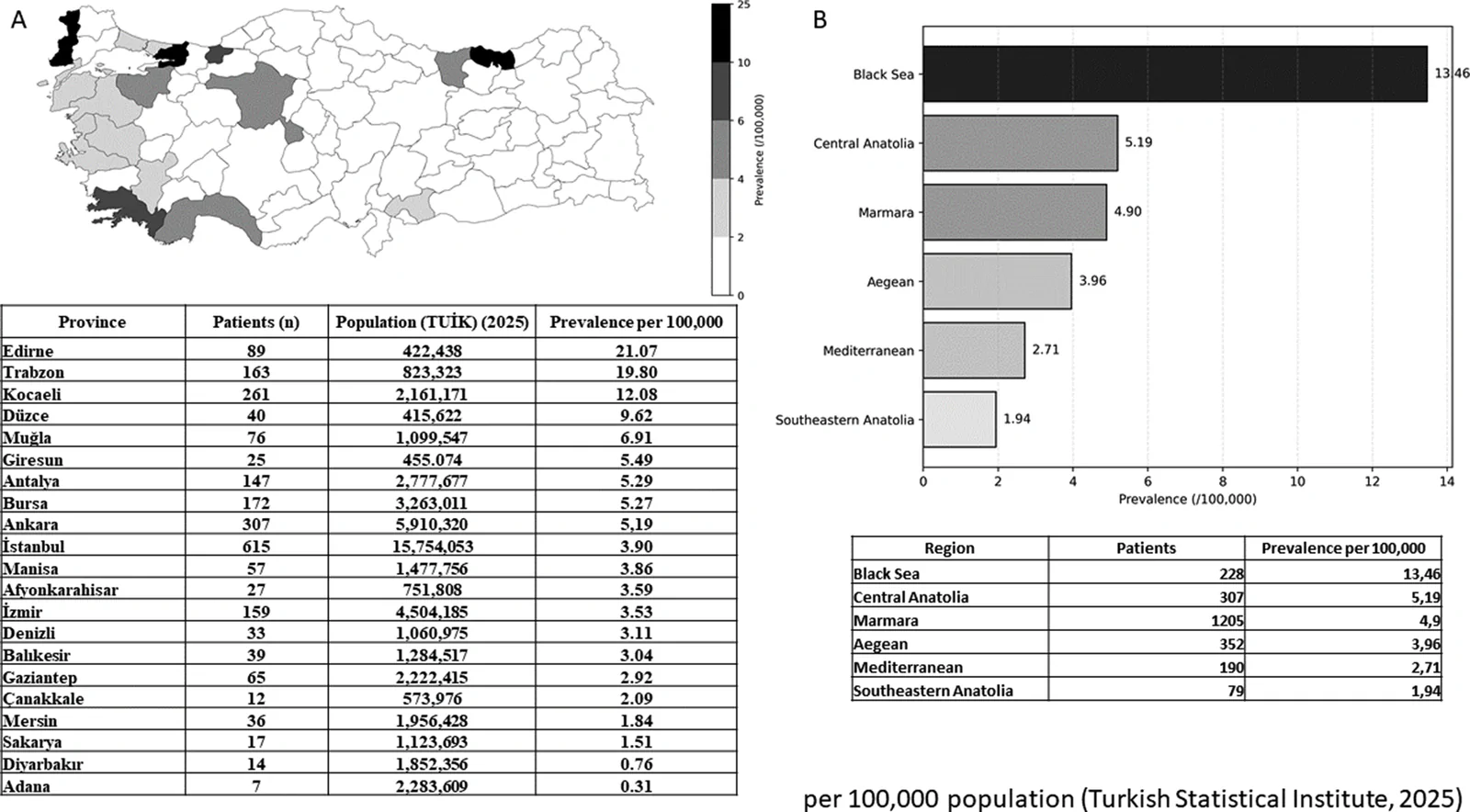
Geographic distribution of study-based ADPKD prevalence in Türkiye. (**A**) Province-level study-based prevalence of ADPKD per 100,000 population using Turkish Statistical Institute (TÜİK) data. **(B)** Regional study-based prevalence estimates calculated using the aggregate population of provinces represented in the cohort within each geographical region

Study-based prevalence analyses demonstrated substantial geographic heterogeneity across Türkiye (Fig. 1). Province-level prevalence ranged from 0 to 21.1 per 100,000 population, with a median prevalence of 3.86 per 100,000 (IQR 2.92–5.49) among provinces reporting cases. Higher prevalence estimates were observed in northwestern and Black Sea provinces, including Kocaeli, Bursa, and Trabzon, whereas lower prevalence was observed in southeastern regions.

At the regional level, the highest study-based prevalence was observed in the Black Sea region (13.46 per 100,000), followed by Central Anatolia (5.19 per 100,000) and Marmara (4.90 per 100,000), whereas lower prevalence estimates were identified in the Mediterranean and Southeastern Anatolia regions (Fig. 1).

**Fig. 2:**
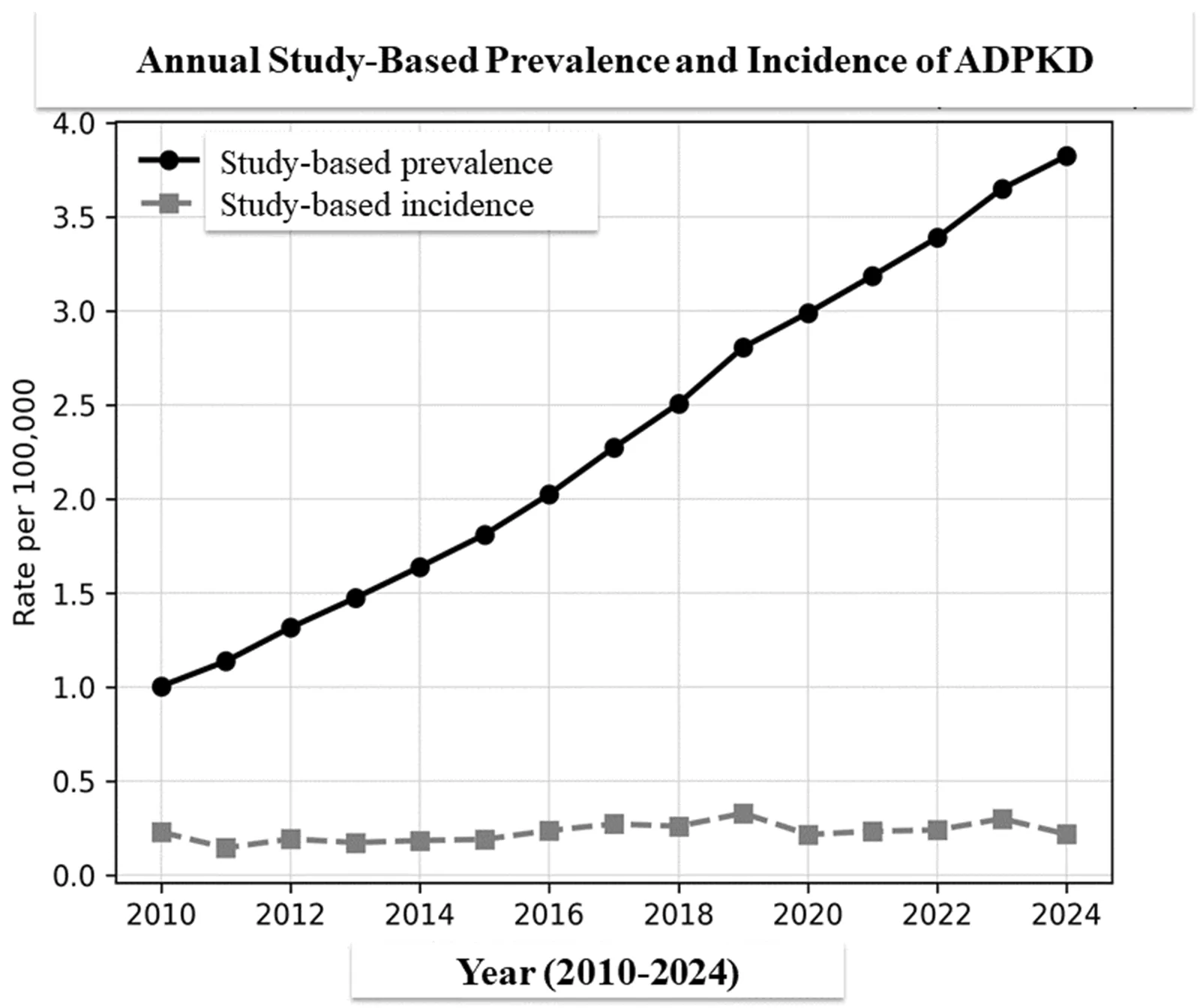
Temporal Trends in study-based incidence and prevalence of ADPKD (2010–2024). Annual study-based incidence and prevalence estimates per 100,000 population calculated using year-specific Turkish Statistical Institute (TÜİK) population data. Temporal trends were assessed using log-linear regression analysis, and annual percent change (APC) values are presented with 95% confidence intervals

Temporal analyses demonstrated a significant increase in study-based prevalence over time, with an annual percent change (APC) of 14.4% (95% CI 13.1–15.6; *p* < 0.001). The increase was more pronounced during earlier years (APC 20.8%) and subsequently plateaued, although prevalence continued to rise throughout follow-up (Fig. 2; Table 2). Annual incidence rates showed year-to-year variability but were generally higher in more recent years than in earlier periods (Fig. 2; Table 2).

**Fig. 3:**
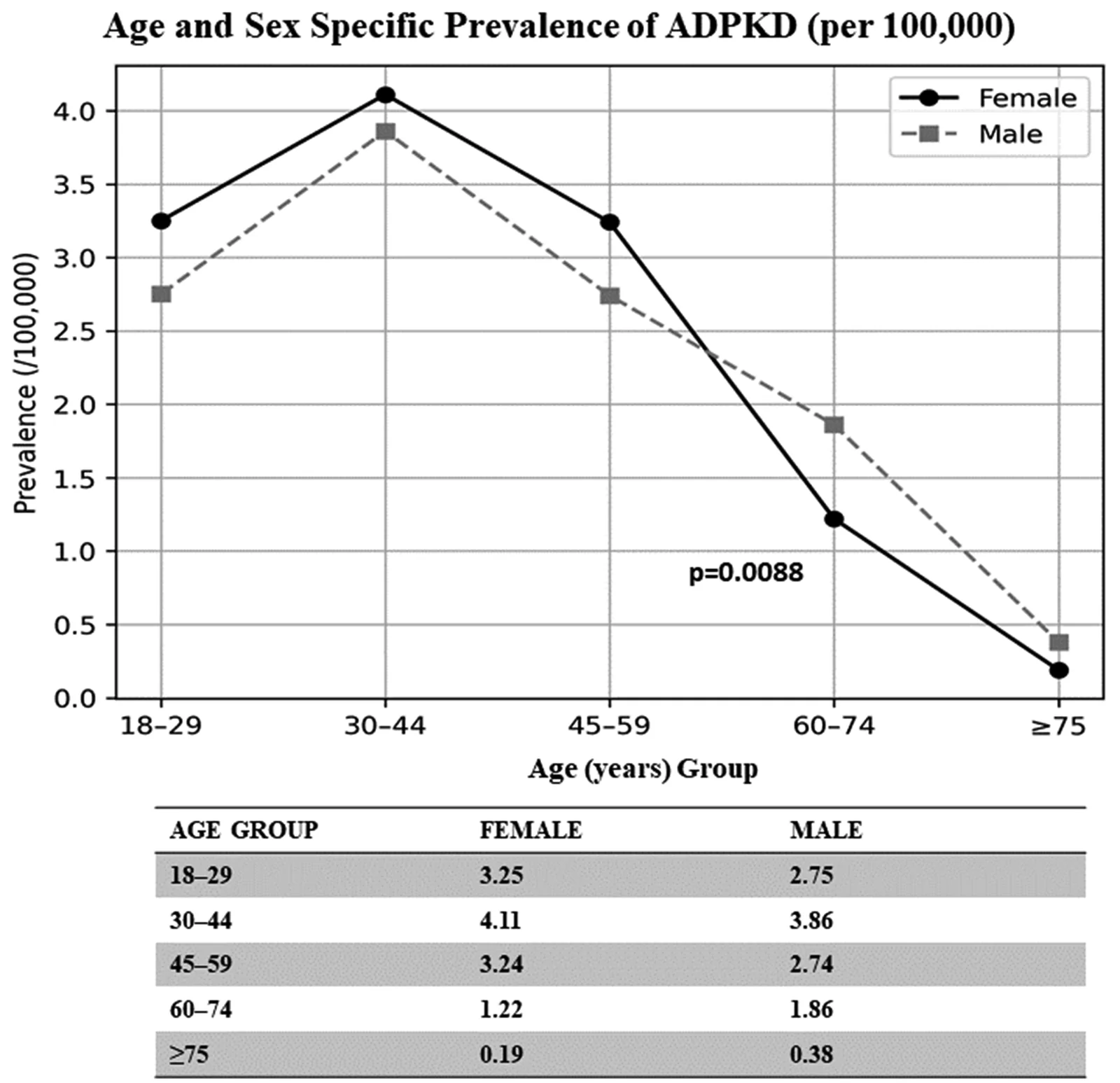
Age- and sex-specific study-based prevalence of ADPKD. Study-based prevalence estimates stratified by age group and sex, expressed per 100,000 population using 2025 estimates from the Turkish Statistical Institute (TÜİK). Prevalence peaked in the 30–44-year age group in both females (4.11 per 100,000) and males (3.86 per 100,000), and declined with advancing age. A statistically significant difference in age distribution between sexes was observed (p=0.0088), with female predominance in younger and middle-aged groups

Age- and sex-specific prevalence analyses demonstrated peak prevalence in the 30–44-year age group in both sexes (4.11 per 100,000 in females and 3.86 per 100,000 in males), followed by a gradual decline with advancing age. Although prevalence patterns were generally similar between sexes, female predominance was observed in younger and middle-aged groups (*p* = 0.0088) (Fig. 3).

### Demographic and clinical characteristics

The mean age at diagnosis was 38.6 ± 14 years, and 51.8% of patients were female (Table 1). The highest number of patients was observed in the 30–44 age group for both sexes (394 males and 419 females), followed by the 18–29 age group (257 males and 304 females). In all age groups, the number of female patients was higher than that of males.

Hypertension was the most common comorbidity and was present in 69.8% of patients, whereas diabetes mellitus and cardiovascular disease were observed in 7.5% and 8.5%, respectively. Renin–angiotensin system blocker therapy was used in 63.6% of patients (Table 1).

Among patients with available Mayo Imaging Classification data, approximately half were classified as high risk (class 1D–1E, 49.6%), whereas 25.6% and 24.8% were categorized as moderate-risk (1 C) and low-risk (1 A–1B), respectively.

Tolvaptan was used in 617 (26.1%), primarily among patients classified as high risk according to the Mayo classification.

### Renal function and longitudinal kidney outcomes

At baseline, median serum creatinine was 0.95 mg/dL (IQR 0.71–1.13), and median eGFR was 94 mL/min/1.73 m² (IQR 60.5–114.5). Median follow-up duration was 6 years (IQR 2–12).

**Fig. 4:**
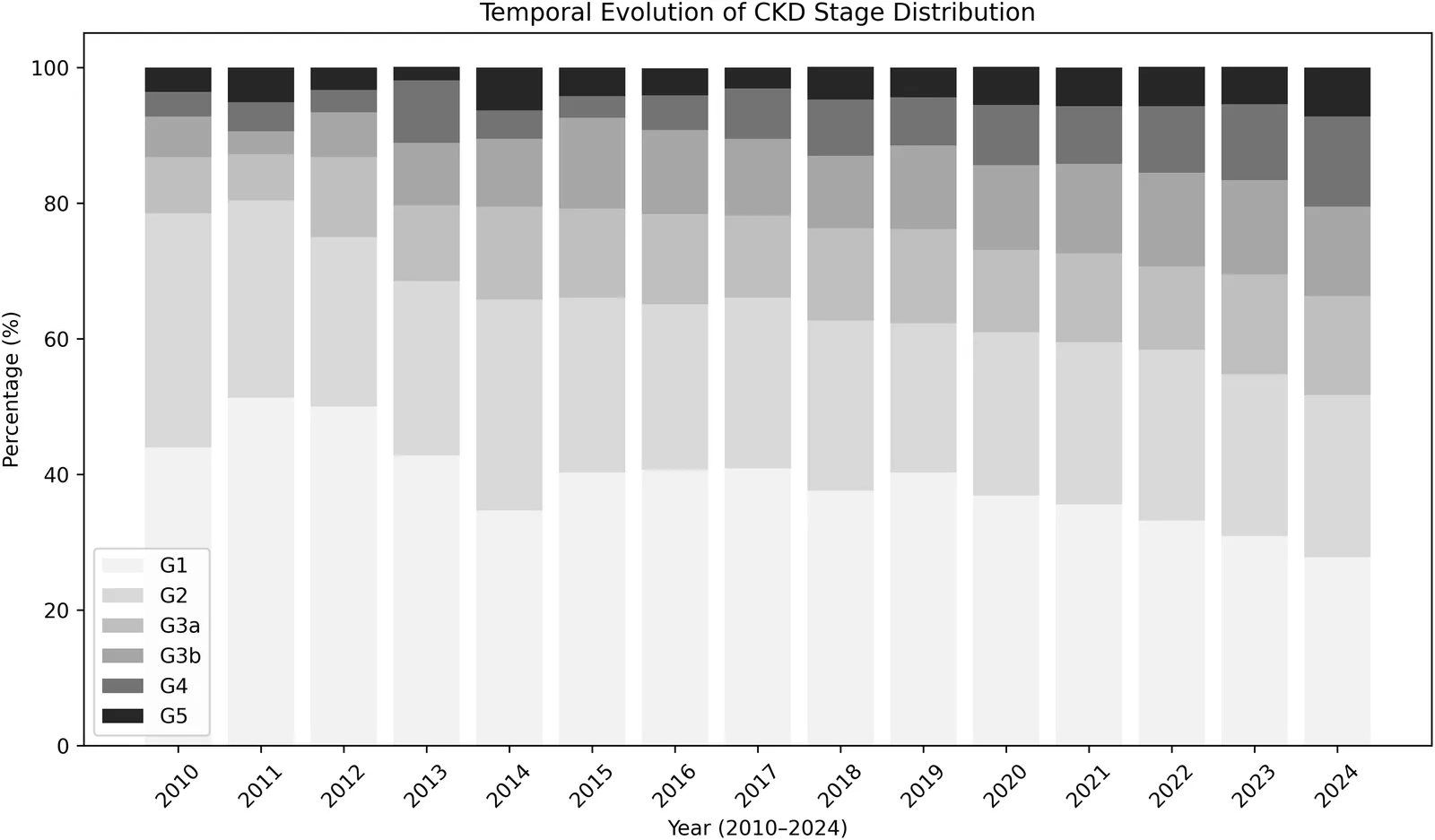
Temporal evolution of CKD stage distribution in ADPKD. Distribution of chronic kidney disease (CKD) stages from 2010 to 2024 according to KDIGO classification [G1 (≥ 90), G2 (60–89), G3a (45–59), G3b (30–44), G4 (15–29), and G5 (< 15 mL/min/1.73 m²)]. Values are expressed as percentages for each calendar year. A progressive shift from early-stage disease (G1–G2) toward advanced stages (G3–G5) is observed over time, consistent with longitudinal decline in renal function (p for trend < 0.001)

Longitudinal analyses demonstrated progressive deterioration in kidney function over time, with increasing serum creatinine levels and declining eGFR values throughout follow-up (*p* < 0.001 for both). A marked temporal shift toward advanced CKD stages was observed, with the proportion of patients classified as G1 decreasing from 44.0% in 2010 to 27.8% in 2024, whereas G3–G5 stages increased progressively over time (p for trend < 0.001) (Fig. 4; Table 3).

**Fig. 5:**
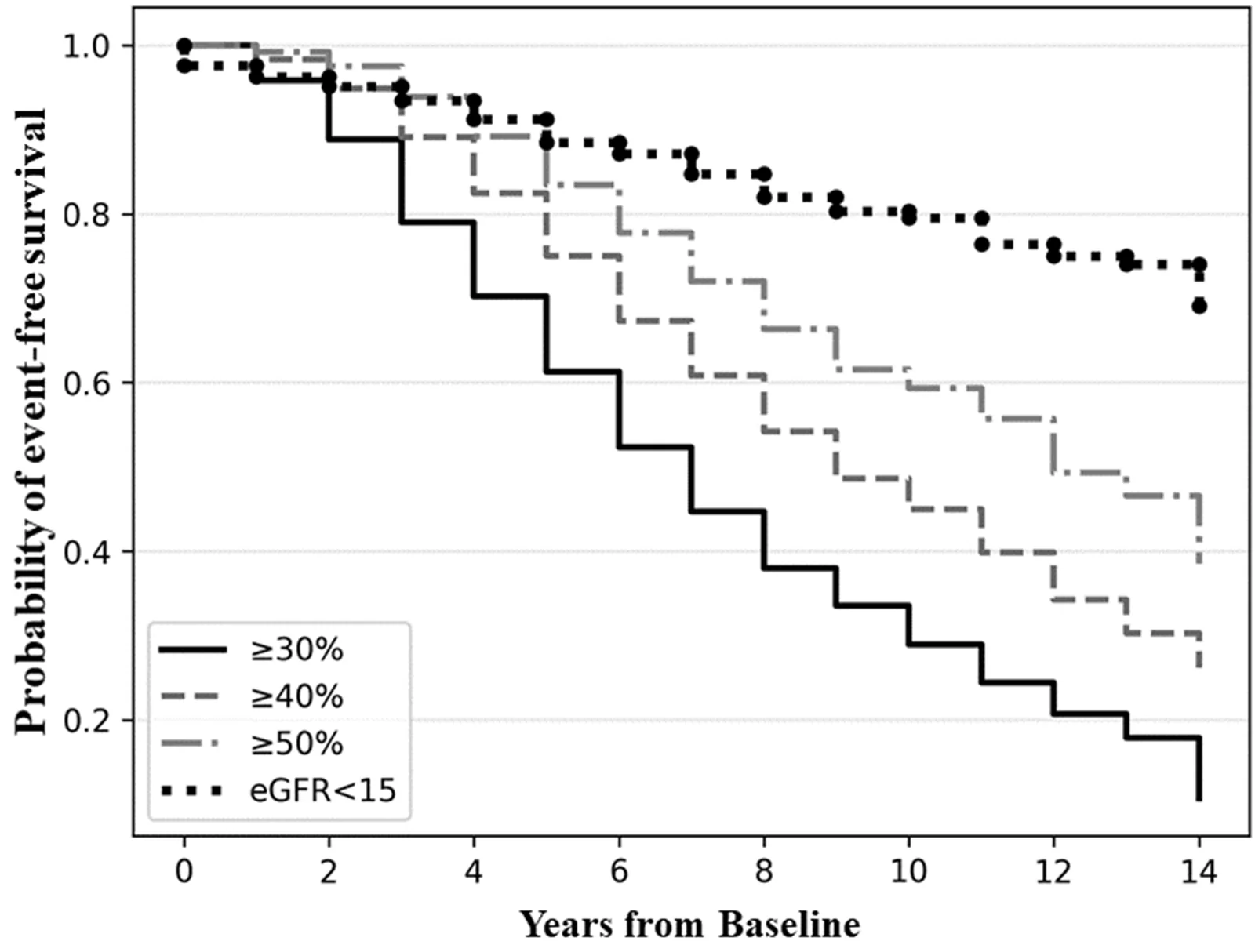
Kaplan–Meier analysis of renal progression in ADPKD. Kaplan–Meier curves showing event-free survival for renal progression endpoints defined as ≥ 30%, ≥ 40%, and ≥ 50% decline in eGFR, and progression to kidney failure (eGFR < 15 mL/min/1.73 m²). Time is measured from baseline eGFR. A stepwise increase in cumulative event rates is observed across endpoints, with earlier separation of curves for less stringent thresholds

Median annual eGFR decline was − 3.33 mL/min/1.73 m²/year (IQR − 5.75 to − 1.25). During follow-up, 34.6% of patients experienced ≥ 30% decline in eGFR, 23.9% experienced ≥ 40% decline, and 16.5% experienced ≥ 50% decline from baseline kidney function. Progression to kidney failure (eGFR < 15 mL/min/1.73 m²) occurred in 10.5% of patients. Kaplan–Meier analyses demonstrated progressive reduction in event-free survival across renal endpoints over time (Fig. 5).

Among patients who progressed to kidney failure (eGFR < 15 mL/min/1.73 m²), age characteristics were broadly comparable across geographical regions with mean age at the last available follow-up ranged from 55.9 years in the Marmara region to 59.6 years in the Aegean region (Supplementary Table S1).

### Imaging and genetic characteristics

Ultrasonography was the most frequently used imaging modality (*n* = 1266, 53.6%). Among patients with available MRI or CT-based TKV measurement (*n* = 1095, 46.4%), 49.6% were classified as high risk (1D, 33.1; %, 1E,16.5%), 25.6% moderate risk (class 1 C), and 24.8% as low-risk (1 A, 7.4%;1B, 17.4%) according to the Mayo classification (Table 1).

Genetic testing information was available in 314 patients; however, definitive genotype classification (*PKD1* or *PKD2*) could only be confirmed in a subset of patients. Among patients with confirmed genotype classification, *PKD1* and *PKD2* mutations were identified at similar frequencies (49% and 51%, respectively).

### Predictors of renal progression

In univariate Cox regression analyses, increasing age (HR per year, 1.02; 95% CI, 1.015–1.027; *p* < 0.001), higher BMI (HR, 1.02; 95% CI, 1.004–1.03; *p* = 0.013), hypertension (HR, 2.56; 95% CI, 1.96–3.35; *p* < 0.001) and cardiovascular disease (HR, 1.62; 95% CI, 1.27–2.07; *p* < 0.001) were associated with increased risk of ≥ 40% eGFR decline. In contrast, RAS blocker use (HR, 0.63; 95% CI, 0.51–0.77; *p* < 0.001) and *PKD2* genotype (HR, 0.83; 95% CI, 0.77–0.89; *p* < 0.001) were associated with lower risk of renal progression (Table 4). Higher Mayo Imaging Classification were associated with progressively greater risk of renal progression. Compared with Mayo class 1 A, classes 1B (HR, 2.19; 95% CI, 1.32–3.61; *p* = 0.002), 1 C (HR, 2.12; 95% CI, 1.29–3.48; *p* = 0.003), and 1D (HR, 3.10; 95% CI, 1.79–5.37; *p* < 0.001) were significantly associated with higher risk. Although Mayo class 1E showed an increased HR, this association did not reach statistical significance (HR, 1.09; 95% CI, 0.50–2.34; *p* = 0.838). Tolvaptan use showed a trend toward reduced risk in univariate analysis (HR, 0.82; 95% CI, 0.67–1.01; *p* = 0.056) (Table 4).

In multivariable analyses, older age (adjusted HR 1.02; 95% CI 1.00–1.03; *p* = 0.010) and hypertension (adjusted HR 2.33; 95% CI 1.25–4.32; *p* = 0.007) remained independently associated with renal progression. Importantly, tolvaptan exposure was independently associated with a lower risk of ≥ 40% eGFR decline in eGFR (adjusted HR, 0.56; 95% CI, 0.37–0.85; *p* = 0.004).

Mayo Imaging Classification retained independent prognostic significance in adjusted analyses (overall *p* = 0.027). Compared with Mayo class 1 A, patients in class 1B (adjusted HR, 1.95; 95% CI, 1.15–3.31; *p* = 0.014) and class 1D (adjusted HR, 2.48; 95% CI, 1.29–4.75; *p* = 0.006) had significantly higher risks of renal progression. Although class 1 C showed an increased HR, this did not reach statistical significance (adjusted HR, 1.55; 95% CI, 0.85–2.83; *p* = 0.147). Other variables, including BMI, sex, cardiovascular disease, RAS inhibitor use, and genotype, were not independently associated with renal outcome in the adjusted model (Table 4).

Exploratory analyses among patients with confirmed *PKD1* or *PKD2* mutations demonstrated numerically higher rates of ≥ 40% eGFR decline among *PKD1* mutation carriers (31.4% vs. 16.2%), whereas progression to kidney failure (eGFR < 15 mL/min/1.73 m²) occurred at similar rates in both groups. However, these differences did not reach statistical significance, likely because of the limited number of genetically characterized patients (Supplementary Table S2).

## Discussion

In this large, multicenter cohort study, we provide a comprehensive evaluation of the study-based epidemiology and clinical outcomes of ADPKD across participating nephrology centers in Türkiye. Several important findings emerge from our analysis. First, the observed study-based prevalence of ADPKD was substantially lower than expected based on genetic estimates, suggesting considerable underrecognition of ADPKD at the population level. Second, we identified marked regional heterogeneity together with a consistent temporal increase in prevalence over time. Third, renal disease progression was substantial despite the relatively young age of the cohort, with a considerable proportion of patients experiencing clinically meaningful decline in kidney function. Finally, tolvaptan exposure was independently associated with a lower risk of renal progression in a real-world clinical setting.

The median province-level prevalence observed in our study (3.86 per 100,000) was markedly lower than both genetic prevalence estimates (1:500–1:2500) [5] and clinically reported prevalence estimates from Europe and North America, which generally range from 25 to 68 per 100,000 [17, 18]. Recent large-scale sequencing and registry-based studies suggest that the true prevalence of ADPKD may be substantially higher than clinically recognized, with estimates approaching 1 in 1000 individuals [19]. Even in well-established healthcare systems, registry-based prevalence estimates remain lower than expected, supporting the possibility that a substantial proportion of affected individuals remain unrecognized [20]. In this context, the relatively low study-based prevalence observed in our cohort likely reflects multiple contributing factors, including limited disease recognition, reliance on ICD-based case identification, and referral bias inherent to tertiary care–based datasets. In addition, the higher prevalence observed in more urbanized regions likely reflects differences in healthcare access, diagnostic imaging availability, and physician awareness rather than true biological differences in disease distribution. A previous multicenter study from Türkiye further demonstrated that nearly one in five patients remained unaware of the hereditary nature of the disease, highlighting an important gap in disease awareness and patient education [21].

The significant increase in prevalence over time (APC 14.4%) further supports this interpretation. Similar temporal trends have been reported in registry-based studies, where increasing prevalence has largely been attributed to improved disease recognition, broader use of imaging modalities, and increasing diagnostic awareness rather than true changes in disease incidence [17, 22]. The deceleration observed during later years in our cohort may reflect partial saturation of diagnostic capture after an initial period of increased recognition.

Renal disease progression was substantial in this cohort, with nearly one-quarter of patients experiencing a ≥ 40% decline in eGFR and more than 10% progressed to kidney failure during follow-up. We selected a ≥ 40% decline in eGFR as the primary endpoint because it is a validated surrogate marker of clinically meaningful kidney disease progression [16]. Given the relatively young age of the cohort and the prolonged disease trajectory of ADPKD, this intermediate endpoint enabled more reliable assessment of longitudinal renal progression during follow-up.

Increasing age and hypertension emerged as independent predictors of renal progression, consistent with previous studies demonstrating the major contribution of demographic and hemodynamic factors to ADPKD progression [19, 23].

Hypertension, in particular, remained one of the strongest independent predictors of adverse renal outcomes, emphasizing its central role in ADPKD pathophysiology and its importance as a modifiable risk factor [23, 24]. These findings further support the need for early and aggressive blood pressure control in patients with ADPKD. Although ACE inhibitor or angiotensin receptor blocker use appeared protective in univariable analyses, this association was attenuated after multivariable adjustment, likely reflecting confounding by indication and differences in baseline disease severity. This protective association is biologically plausible and supported by prior evidence on RAAS blockade in ADPKD, our finding highlights the importance of cautious interpretation of treatment effects in observational studies [25, 26].

Genetic testing was available in only a limited subset of patients, highlighting persistent barriers to routine genetic evaluation in real-world clinical practice despite its recognized prognostic importance [5]. This limited availability restricted comprehensive genotype–phenotype analyses within our cohort. Expanding access to genetic testing, together with established imaging-based tools such as the Mayo Imaging Classification, may improve prognostic assessment and individualized risk stratification in ADPKD [13].

The Mayo Imaging Classification remained independently associated with renal outcomes, further supporting its value as a robust prognostic tool in real-world clinical settings [13, 14, 27]. Although the association across subclasses was not strictly linear, higher Mayo classes generally corresponded to increased risk of renal progression. The lack of statistical significance observed for class 1E despite higher point estimates may reflect limited sample size, treatment-related modification of risk, or heterogeneity within advanced disease stages, as frequently encountered in observational cohorts [13, 20].

One of the most clinically relevant findings of our study is the independent association between tolvaptan exposure and lower risk of renal progression (adjusted HR 0.56). This finding is consistent with randomized controlled trials, including the TEMPO 3:4 and REPRISE studies, which demonstrated that vasopressin V2 receptor antagonism slows the rate of kidney function decline in patients with ADPKD [27, 28]. Importantly, our results extend these observations to a real-world setting and suggest that the benefits observed in clinical trials may also be reproducible in routine clinical practice. Although residual confounding cannot be excluded because of the observational design, the persistence of this association after adjustment for clinical and imaging-based risk factors supports the effectiveness of tolvaptan in patients at risk of rapid disease progression. The detailed analyses of tolvaptan-treated patients were beyond the scope of this study.

Several limitations should be acknowledged. First, prevalence estimates were study-based and derived from selected provinces represented in participating centers, which may limit generalizability. Additionally, the absence of data from certain regions, such as Eastern Anatolia, may have affected the overall geographic distribution. Second, case identification relied on ICD coding and may therefore have resulted in misclassification or incomplete case ascertainment, particularly in asymptomatic or early-stage disease. Third, the retrospective design and tertiary-center recruitment may have introduced selection bias toward more advanced disease. Fourth, genetic data were unavailable in a substantial proportion of patients, limiting genotype-based analyses. Finally, missing data were handled using a complete-case approach, which may introduce bias if data were not missing at random.

In conclusion, the PKD-TURK cohort provides one of the largest real-world evaluations of ADPKD in Türkiye, integrating epidemiological analyses with longitudinal renal outcomes. The relatively low study-based prevalence suggests substantial underrecognition of ADPKD, whereas marked regional variation highlights disparities in disease recognition and healthcare access. The considerable burden of renal progression observed in this relatively young cohort underscores the need for earlier diagnosis and improved risk stratification strategies. In addition, the association between tolvaptan exposure and lower risk of renal progression further supports its effectiveness in routine clinical practice. Collectively, these findings provide important real-world insights into the epidemiological and clinical landscape of ADPKD in an underrepresented population and may help inform future healthcare planning and early intervention strategies.

**Table 1:** Demographic and clinical characteristics of the study population

	*n* = 2361
Age, years (mean ± sd)	38.6 ± 14
Sex, n (%)	
Female Male	1221 (51.8) 1140 (48.2)
Anthropometric characteristics	
Height (cm) Weight (kg) BMI (kg/m^2^)	168(161, 175) 75 (67, 85) 26.2 (23.9, 29.2)
Geographical Region, n(%)	
Marmara Aegean Central Anatolia Black Sea Mediterranean Southeastern Anatolia	1205 (51) 353 (15) 307 (13) 228 (10) 190 (8) 78 (3)
Comorbidities, n (%)	
Hypertension Diabetes Mellitus Cardiovascular Disease	1647 (69.8) 178 (7.5) 201 (8.5)
RAS Blocker Use, n (%)	1502 (63.6)
Tolvaptan Exposure, n (%)	617 (26.1)
ADPKD Characteristics	
Follow-up duration, years (IQR)	6 (2, 12)
Baseline serum Creatinine (mg/dL)	0.95 (0.71,1.13)
Baseline eGFR (ml/min/1.73 m2)	94 (60.5, 114.5)
MIC class, n (%)	
1 A 1B 1 C 1D 1E	82 (7.4) 190 (17.4) 280 (25.6) 362 (33.1) 181 (16.5)

SD, standard deviation; IQR, interquartile range; BMI, body mass index; RAS, renin–angiotensin system; ADPKD, autosomal dominant polycystic kidney disease; eGFR, estimated glomerular filtration rate; MIC, Mayo Imaging Classification. Baseline values refer to measurements obtained at the time of ADPKD diagnosis. Baseline values are presented as mean ± SD, median (IQR), or n (%), as appropriate

**Table 2:** Annual study-based incidence and prevalence estimates of ADPKD in Türkiye

Year	Year-Specific Adult population by TUIK	Incidence per 100,000 population	Prevalence per 100,000 Population
2010	43,626,067	0.229	1.004
2011	44,149,579	0.145	1.137
2012	44,679,374	0.192	1.316
2013	45,215,527	0.173	1.473
2014	45,758,113	0.184	1.639
2015	46,307,211	0.190	1.810
2016	46,862,897	0.237	2.025
2017	47,425,252	0.272	2.273
2018	47,994,355	0.260	2.507
2019	48,570,287	0.329	2.806
2020	49,153,131	0.216	2.989
2021	49,742,968	0.233	3.186
2022	50,339,884	0.240	3.389
2023	50,943,962	0.300	3.649
2024	51,555,290	0.217	3.823
2025	52,173,954	0.048	3.826

Incidence was defined as the number of newly diagnosed ADPKD cases per calendar year, and prevalence as cumulative diagnosed cases. Rates are expressed per 100,000 population based on Turkish Statistical Institute data. Data for 2025 include the first 6 months only. ADPKD, autosomal dominant polycystic kidney disease; TUIK, Turkish Statistical Institute

**Table 3:** Annual median serum creatinine, eGFR, and distribution of CKD stages in the study population

Years	Creatinine, mg/dL (median, IQR)	eGFR, mL/min/1.73 m^2^ (median, IQR)	CKD Stages					
			G1(%)	G2 (%)	G3a (%)	G3b (%)	G4(%)	G5(%)
2010	0.95 (0.71,1.13)	94 (64.7, 112.7)	44.0	34.5	8.3	6.0	3.6	3.6
2011	0.87 (0.70, 1.2)	91 (60.5, 114.5)	51.3	29.1	6.8	3.4	4.3	5.1
2012	0.81 (0.70, 1.2)	89 (59.3, 112)	50.0	25.0	11.8	6.6	3.3	3.3
2013	0.91(0.73, 1.31)	82 (50.5, 107.5)	42.8	25.7	11.2	9.2	9.2	2.0
2014	0.97 (0.75, 1.44)	79 (48.7,100.4)	34.7	31.1	13.7	10.0	4.2	6.3
2015	1.00 (0.74, 1.45)	81 (49.0, 107)	40.3	25.8	13.1	13.4	3.2	4.2
2016	1.00 (0.73, 1.42)	79 (47.7, 107)	40.7	24.4	13.3	12.4	5.1	4.0
2017	1.03 (0.76, 1.57)	79 (48.0, 108)	40.9	25.2	12.1	11.3	7.4	3.1
2018	1.08 (0.77, 1.66)	75 (45.0, 108)	37.6	25.1	13.6	10.7	8.3	4.8
2019	1.09 (0.77, 1.64)	74 (46.0, 108)	40.3	22.0	13.9	12.3	7.1	4.4
2020	1.12 (0.79, 1.80)	73 (43.0,106.6)	36.9	24.1	12.1	12.5	8.9	5.6
2021	1.21 (0.82, 2.46)	71 (42.0, 102.2)	35.6	23.9	13.1	13.2	8.5	5.7
2022	1.19 (0.85, 1.9)	68 (40.1, 100)	33.2	25.2	12.3	13.8	9.8	5.8
2023	1.23 (0.86, 1.98)	65 (38.0, 100)	30.9	23.9	14.7	13.9	11.2	5.5
2024	1.30 (0.91, 2.19)	62 (35.8, 93.5)	27.8	23.9	14.6	13.2	13.3	7.2

Values are presented as median (interquartile range) or percentages, as appropriate. CKD stages were defined according to KDIGO criteria as follows: G1 (≥ 90), G2 (60–89), G3a (45–59), G3b (30–44), G4 (15–29), and G5 (< 15 mL/min/1.73 m²). Percentages represent the distribution of CKD stages for each calendar year. Temporal trends were assessed using the Cochran–Armitage test (p for trend < 0.001). eGFR, estimated glomerular filtration rate; CKD, chronic kidney disease; KDIGO, Kidney Disease: Improving Global Outcomes

**Table 4:** Univariable and multivariable Cox proportional hazards analyses for ≥ 40% decline in eGFR

Variable	Univariate analysis			Multivariable analysis		
	HR	95% CI	*p*-value	HR	95% CI	*p*-value
Age (per year)	1.02	1.015–1.027	< 0.001	1.02	1.00–1.03	0.010
Male sex	1.10	0.92–1.31	0.30	0.86	0.63–1.18	0.346
BMI	1.02	1.004–1.03	0.013	1.01	0.99–1.03	0.396
Genotype (*PKD2* vs. *PKD1*)	0.83	0.77–0.89	< 0.001	-	-	-
Diabetes mellitus	1.07	0.79–1.43	0.68	-	-	-
Hypertension	2.56	1.96–3.35	< 0.001	2.33	1.25–4.32	0.007
Cardiovascular disease	1.62	1.27–2.07	< 0.001	1.16	0.73–1.83	0.535
ACEi/ARB use	0.63	0.51–0.77	< 0.001	1.23	0.73–2.05	0.437
Tolvaptan use	0.82	0.67–1.01	0.056	0.56	0.37–0.85	0.004
Mayo classification (overall)				-	-	0.027
Mayo class 1B vs. 1 A	2.19	1.32–3.61	0.002	1.95	1.15–3.31	0.014
Mayo class 1 C vs. 1 A	2.12	1.29–3.48	0.003	1.55	0.85–2.83	0.147
Mayo class 1D vs. 1 A	3.10	1.79–5.37	< 0.001	2.48	1.29–4.75	0.006
Mayo class 1E vs. 1 A	1.09	0.50–2.34	0.838	1.17	0.48–2.86	0.736

Hazard ratios (HRs) are presented with 95% confidence intervals (CIs). Reference categories were female sex, *PKD1* genotype, absence of diabetes mellitus, absence of hypertension, absence of cardiovascular disease, no use of ACE inhibitors or angiotensin receptor blockers, no tolvaptan use, and Mayo class 1 A. Age was analyzed as continuous variables. Variables included in the multivariable model were selected based on clinical relevance and univariable analysis (*p* < 0.10). HR, hazard ratio; CI, confidence interval; BMI, body mass index; ACEi, angiotensin-converting enzyme inhibitors; ARB, angiotensin receptor blockers

## Supplementary Information

Below is the link to the electronic supplementary material.

Supplementary Material 1

Supplementary Material 2

## Data Availability

The datasets generated and/or analyzed during the current study are available from the corresponding author upon reasonable request.

## Abbreviations

ADPKD: Autosomal dominant polycystic kidney disease
APC: Annual percent change
BMI: Body mass index
CI: Confidence interval
CKD: Chronic kidney disease
CKD-EPI: Chronic Kidney Disease Epidemiology Collaboration
eGFR: Estimated glomerular filtration rate
ESKD: End-stage kidney disease
HR: Hazard ratio
htTKV: Height-adjusted total kidney volume
ICD-10: International Classification of Diseases, Tenth Revision
IQR: Interquartile range
KDIGO: Kidney Disease: Improving Global Outcomes
MRI: Magnetic resonance imaging
CT: Computed tomography
RAS: Renin–angiotensin system
TÜİK: Turkish Statistical Institute
