# Supplementary material for: Real-world epidemiology and renal outcomes of ADPKD in Türkiye: the PKD-TURK cohort
Source: BMC Nephrol. 2026 Jul 24;27:622. doi: 10.1186/s12882-026-05217-0 (PMC13685119; doi:10.1186/s12882-026-05217-0)
Supplement: Supplementary file 1 [file 12882_2026_5217_MOESM1_ESM.docx]

| **Region** | **n** | **Mean age at last visit (years)** |
| --- | --- | --- |
| Marmara | 84 | 55.9 ± 11.8 |
| Aegean | 20 | 59.6 ± 11.3 |
| Central Anatolia | 15 | 57.4 ± 9.5 |
| Black Sea | 44 | 59.3 ± 12.4 |
| Mediterranean | 11 | 56.6 ± 12.6 |
| Southeastern Anatolia | 1 | 43 |
| Total | 175 | 57.3 ± 11.8 |

**Supplementary Table S1: Age characteristics of patients reaching kidney failure (eGFR <15 mL/min/1.73 m²) across geographical regions**

*Age was calculated at the last available follow-up among patients who had reached kidney failure (eGFR <15 mL/min/1.73 m²).
