# Supplementary material for: Real-world epidemiology and renal outcomes of ADPKD in Türkiye: the PKD-TURK cohort
Source: BMC Nephrol. 2026 Jul 24;27:622. doi: 10.1186/s12882-026-05217-0 (PMC13685119; doi:10.1186/s12882-026-05217-0)
Supplement: Supplementary file 2 [file 12882_2026_5217_MOESM2_ESM.docx]

| **Outcome** | **PKD1** | **PKD2** | **p value** |
| --- | --- | --- | --- |
| ≥40% eGFR decline | 11/35 (31.4%) | 6/37 (16.2%) | 0.129 |
| eGFR <15 mL/min/1.73m² | 5/39 (12.8%) | 5/39 (12.8%) | 1.000 |

**Supplementary Table S2: Exploratory renal outcome analyses according to genotype among patients with confirmed PKD1 or PKD2 mutations**
